# Lysyl hydroxylases are transcription targets for GATA3 driving lung cancer cell metastasis

**DOI:** 10.1038/s41598-018-30309-9

**Published:** 2018-08-09

**Authors:** Wei Liu, Ting Zhang, Lixia Guo, Yuanyuan Wang, Yanan Yang

**Affiliations:** 1 Thoracic Disease Research Unit, Division of Pulmonary and Critical Care Medicine, College of Medicine and Science, Mayo Clinic, Rochester, Minnesota USA; 2 Department of Respiratory, the Second Affiliated Hospital, the Fourth Military Medical University, Xi’an, ShaanXi China; 3 Department of Respiratory Medicine, East Hospital, Tongji University School of Medicine, Shanghai, China; 4 Department of Biochemistry and Molecular Biology, College of Medicine and Science, Mayo Clinic, Rochester, Minnesota USA; 5 Developmental Therapeutics and Cell Biology programs, Mayo Clinic Cancer Center, Rochester, Minnesota USA

## Abstract

Metastasis associates with late stages of lung cancer progression and remains the main cause of patient death due to the lack of clinically effective therapeutics. Here we report that the transcription factor GATA3 and its co-factor FOG2 commonly promote the expression of the lysyl hydroxylase (LH) family members, including LH2 and LH3, which in turn drive lung adenocarcinoma cell migration, invasion, and metastasis. We show evidence that both LH2 and LH3 are direct transcription targets for GATA3. Knockdown of either LH2 or LH3 suppresses migration and invasion; on the contrary, forced expression of LH2 or LH3 promotes growth and migration, suggesting that the two LHs exert redundant oncogenic functions. Importantly, re-expression of LH2 is sufficient to restore the metastatic capacity of GATA3-depleted cells, suggesting a role for LHs as the downstream mediators of GATA3. Collectively, our data reveal a pro-metastatic GATA3-LHs axis for lung cancer, supporting the notion that targeting LHs may be useful for treating lung cancer.

## Introduction

Lung cancer is the deadliest malignancy worldwide and causes ~1.5 million deaths each year according to the WHO statistics (www.who.int). Early detection and treatment have been shown to improve clinical outcomes significantly. However, despite of the continuous improvement of clinical techniques, early detection is only successful for a small population of patients. Most lung cancer patients are diagnosed at late stages, at which the disease has spread to distant sites/organs through metastasis, a multi-step and complicated process enabling invasive cancer cells to disseminate from primary tumors. At least partly due to the incomplete understanding of the biology of metastasis, clinically effective therapies that cure metastasis do not exist, and metastasis has remained the primary cause of lung cancer patient death.

Lysyl hydroxylases are a family of oxygenases catalyzing the hydroxylation of lysine residues on collagen polypeptides, and such lysyl hydroxylation generates crosslinks that are critical for the stability of collagen^1–3^. Although collagen is an important component of extracellular matrix (ECM), which plays critical roles in the regulation of cancer development and malignant progression^4–6^, the role of LHs in cancer metastasis has been poorly understood. Recent studies from us and others have shown that one of the LHs, LH2, may be required for metastasis in several types of cancers, including breast cancer^7^, sarcoma^8^, lung cancer^9^, and renal cell carcinoma^10^, suggesting a potential therapeutic value for LHs. Notably, because LHs may catalyze the hydroxylation of distinct lysine residues on collagen or localize to distinct cellular compartments to exert their biological functions, it is unclear whether other LHs exert similar functions in metastasis.

GATA3 belongs to the GATA family of transcription factors (GATA1-6) that have been involved in the regulation of a variety of biological and pathological processes, ranging from embryonic development to diseases, for instance cancers^11–14^. Previously, we have shown that GATA3 may act as a downstream mediator of the NOTCH ligand JAGGED2 to promote tumor cell migration, invasion, and dissemination, by binding to the promoter and silencing the transcription of microRNA-200 (miR-200), a small non-coding RNA that exerts tumor suppressive functions in KRAS mutant lung cancer cells^15–18^. Consistent with these findings, we found that the friend of GATA 2 (FOG2), a co-factor for GATA factors, is highly expressed by mesenchymal-like lung adenocarcinoma cells, where it drives metastasis^19^. These results lead us to postulate that common transcription targets of FOG2 and GATA3 may be the drivers of metastasis and serve as putative therapeutic targets for treating metastatic lung cancer.

To identify the common targets of FOG2 and GATA3, we performed RNA-sequencing for metastatic lung adenocarcinoma cells expressing shRNAs against FOG2 or GATA3^19,20^. Among the common FOG2- and GATA3-regulated genes, we found that GATA3 promotes the expression of LH2 and LH3 by directly binding to their promoter elements. *In vitro*, forced expression of either LH2 or LH3 promotes lung adenocarcinoma cell growth and migration, and knockdown of LH2 or LH3 suppresses migration and invasion. *In vivo*, re-expression of LH2 in GATA3-depleted cells facilitates their xenograft tumor growth and metastatic capacity. Taken together, our findings suggest that LHs are pro-metastatic mediators of FOG2 and GATA3 and may be useful candidate therapeutic targets for treating lung cancer.

## Materials and Methods

### Cell culture and reagents

Lung adenocarcinoma cancer cell lines, including 393 P, 344SQ, H1299, and HCC827 cells, were cultured in a humidified atmosphere with 5% CO2 at 37 °C in RPMI 1640 medium supplemented with 10% FBS. Anti-flag antibody (clone M2) was purchased from Sigma. Rabbit anti-GATA3 polyclonal antibody (sc-9009) and normal rabbit IgG were purchased from Santa Cruz. Rabbit anti-Tubulin (2125) antibody was purchased from Cell Signal Technology. Human LH2 and LH3 expression plasmids were gifts from Jonathan Kurie MD (the University of Texas MD Anderson Cancer Center). Human and mouse PLOD2 siRNA were purchased from OriGene and Santa Cruz, respectively. Mouse PLOD3 siRNAs were purchased from Santa Cruz. The ChIP essay kit was purchased from ActiveMotif (#53009).

### Cell lysate preparation and Western blotting

Cells were washed by PBS and lysed in a RIPA lysis buffer (Santa Cruz Biotechnologies). Total protein lysates were quantified for protein concentrations with a BCA protein assay kit (Pierce Biotechnology). Protein samples were separated on denaturing SDS–polyacrylamide gel and transferred to a nitrocellulose membranes (Amersham Hybond ECL; GE Healthcare), followed by incubating with appropriate primary antibodies overnight at 4 °C. After being washed with TBST for 30 minutes at room temperature, the membrane was further incubated with HRP-conjugated secondary antibodies for 1 hour, followed by 30 minutes of washing with TBST. Protein bands were visualized with supersignal ECL substrates (Pierce Biotechnology). Uncut western blot gels are shown in Supplemental Fig. 1.

### Cell proliferation assay (MTT assay and soft agar colony formation assay)

For MTT assay, cells (1 × 10^3^ cells/well) were plated in quadruplicate in RPMI 1640 supplemented with 10% FBS on 96-well cell culture plates. Cells were cultured and subjected to a 3-[4,5-dimethylthiazol-2-yl]-2,5 diphenyl tetrazolium bromide (MTT) assay (M5655; Sigma-Aldrich) for 4 hours at 37 °C. Cells were then lysed in dimethyl sulfoxide (200 μl/well), and the optical density at 570 nm was measured on a BenchMark microplate reader (BioRad). For soft agar assay, cells (1 × 10^4^ cells/well were seeded in 0.4% agar in RPMI 1640 10% FBS on 6-well plates as the upper layer, with 0.6% agar in RPMI 1640 10% FBS being the bottom layer. After two weeks, the total number of cell colonies was counted by a Celcount machine (Oxford Optronix, United Kingdom).

### Cell transfection

Transient cell transfection were performed by using lipofectamine 2000 for DNA plasmids or lipofectamine RNAiMAX for siRNAs as described by the manufacturer (Invitrogen). To stably express LH2, the 393 P and GATA3 shRNA-expressing 344SQ lung cancer cells^20^ were transfected with a pEF-bsr-FLAG-LH2 expression plasmid. 48 hours after transfection, cells were prepared into single cell suspension and cultured in the presence of blasticidin (10 μg/ml) for 1 week. To stably express LH3, the 393 P and HCC827 lung cancer cells were transfected with a pLVX-puro2-FLAG-LH3 expression plasmid. 48 hours after transfection, cells were prepared into single cell suspension and cultured in the presence of puromycin (1 μg/ml) for 2 week.

### Chromatin immunoprecipitation assay

ChIP immunoprecipitation assay was performed using the ChIP assay kit according to the manual provided by the manufacturer (ActiveMotif). Briefly, H1299 cells were transiently transfected with GATA3 cDNA for 48 hours and were cross-linked with 1% formaldehyde and then incubated in lysis buffer on ice for 30 minutes. After being grinded several times at 4 °C, the nuclei were pelleted by centrifugation at 5,000 rpm for 10 min at 4 °C. The nuclei were then digested and the sheared chromatin was subjected to immunoprecipitations. For the immunoprecipitations, ChIP Grade anti-GATA3 antibody and normal rabbit anti-IgG antibody were used. Immunoprecipitated DNA was amplified by quantitative real-time PCR (qPCR) using an ABI 7500 fast instrument (Thermo Fisher Scientific Inc).

**Figure 1:**
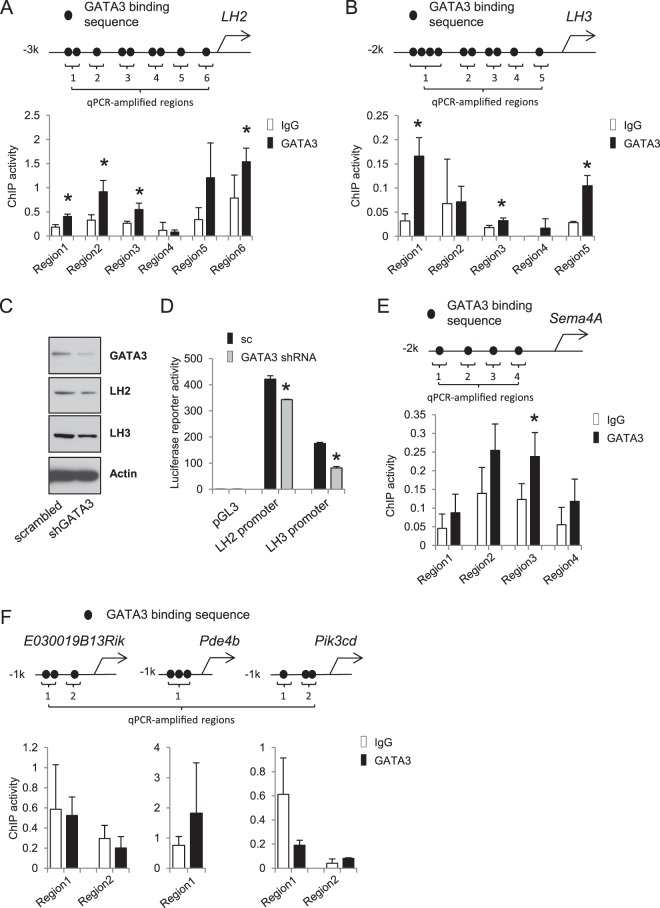
GATA3 regulates gene expression through both direct and indirect mechanisms. (**A**,**B**) GATA3 binds to the promoter elements of LH2 and LH3. Upper: schematic for LH2 (**A**) and LH3 (**B**) promoters containing putative GATA3 binding sites. Bottom: ChIP assay results validating the binding of GATA3 to the promoters. Note: for each of the promoters shown in Fig. 1, qPCR primers were designed to amplify the sequence containing candidate GATA3 binding sites. For promoters containing multiple distant GATA3 sites, multiple pairs of primers were designed to amplify the corresponding promoter regions, which were numbered starting from the most distal region as illustrated in Fig. 1.; in all figures, *indicates t-test p value is less than 0.05. (**C**) Western blots of 393 P cells transfected with scrambled or GATA3 shRNAs. (**D**) Luciferase reporter assays showing that the knockdown of GATA3 inhibited the promoter activities of both LH2 and LH3. (**E**,**F**) ChIP assays for the promoter regions of SEMA4A, E030019B13Rik, Pde4b, and Pik3cd genes as did in (**A**) and (**B**).

For each of the promoters shown in Fig. 1, qPCR primers were designed to amplify the sequence containing candidate GATA3 binding sites. For promoters containing multiple distant GATA3 sites, multiple pairs of primers were designed to amplify the corresponding promoter regions, which were numbered starting from the most distal region as illustrated in Fig. 1. The primers used to amplify the LH2, LH3, Sema4a, E030019B13Rik, Pik3cd and Pde4b promoter elements were as follows: LH2: Region 1. F 5′-TGGTTCAATGAAGGAGGGGTG-3′, R 5′-AGTTAGGTTGGCATGCTCAGAA-3′, Region 2. F 5′-GTTTCATGGCCCTTCCGTCA-3′, R 5′- TAGGGCCTTTAGTCCACAGAC-3′, Region 3. F 5′-TTACTGGACAAACTGCACCCC-3′, R 5′- GACACTGGAGGAGAACAAAGC-3′, Region 4. F 5′-AGAGACAAAGTATGCCAAACCA-3′, R 5′- TGCCCTGGTTCAAGCTTTT-3′, Region 5. F 5′-GAGCCATGTAAGTAAGAGGATCA-3′, R 5′- GGTTTCCAGCCCTCCTGTAG-3′, Region 6. F 5′-TTGCCCCTGCTTGGATTCAT-3′, R 5′- CTGTGATTGCAGAAGAATGGAGT-3′. LH3: Region 1. F 5′-AAAGTCTCCCCAGTGTGGCT-3′, R 5′-ATTGAAGGTTGTGGGGGTGA-3′, Region 2. F 5′-TATCAGCAGGGGCTCATCTCT-3′, R 5′-AGTCCCCAGGATCCACATGAT-3′, Region 3. F 5′-CCAGAAGCTGGAGGAAAGTG-3′, R 5′- GAGCAGTGGTACTCACCTCG-3′, Region 4. F 5′-GGAGATCAGCAAGCTTTCGC-3′, R 5′- GACAAGAGCGCTCCCCTAAA-3′, Region 5. F 5′-TAAAAACTGGCGTGGGTGGT-3′, R 5′- CAACAAAGCCACCGAACCTG-3′. Sema4a: Region 1. F 5′-TCCTCTGGTCACAGCTCCTC-3′, R 5′-CACCTCAGGGCCTCTCTTTG-3′, Region 2. F 5′-TGGCAGTGAGGATGGTGTTT-3′, R 5′- CAGTGACCCAAGCTGGTCTA-3′, Region 3. F 5′-GAAGTGGTGTGCGGGAGAAA-3′, R 5′- CACCTCCCTCCCTCCTTAGT-3′, Region 4. F 5′-CAGGCCACTCCCAGGAAATAC-3′, R 5′- CTTAATCGGGCCCATCTAGCC-3′. E030019B13Rik: Region 1. F 5′- TTCTTCCCCAGCCGGTTTAG-3′, R 5′-TGTAGCTGACACACGTTCCC-3′, Region 2. F 5′- CGGCCATCCACTAGGATAGA-3′, R 5′-GGACCCAGACACCGCTTAC-3′. PIK3cd: Region 1. F 5′-GCATGACCGATATCGTAACTCCT-3′, R 5′-GCAAAGCCAGCCTCGGTTAT-3′, Region 2. F 5′- GGCTCCAATTCAGAGCCTCAAG-3′, R 5′-GCCATCTTCTACTACATTGC-3′. Pde4b: Region 1.F 5′-GTGAGTCTCAGTGTGTAACTTGC-3′, R 5′-CCCAGGCTAATTTGGGAAGC-3′.

### Luciferase-conjugated promoter reporter assay

Luciferase-conjugated human LH2 promoter reporter construct in the pGL3 basic vector (Promeaga) was generated by our previous studies^9^, and we cloned a ~1.5 k human LH3 promoter into the same vector using the following primers: F: 5′-GGGGTACCTCACTGCCTTTGCACGTGAG-3′, and 5′-CGGCTAGCACAGAAAGCTCCAGAT GCCG-3′. Reporter assays were performed as we described^9^. Briefly, cells were grown to 90% confluency in 24-well plates and transfected with the promoter reporter constructs or with the empty pGL3 vector (500 ng per well), and co-transfected with a pRL-TK (Promeaga, 10 ng per well) as internal control. 72 hours after the transfection, the luciferase reporter activities were measured with the Dual-Luciferase assay kit from Promeaga according to the manufacturer’s datasheet.

### Migration and invasion assays

Migration and invasion abilities of tumor cells were quantitated by using 24 well plate 8 μm pore size of noncoated (for migration assay) or matrigel-coated (for invasion assay) transwells as described by the manufacturer (BD Biosciences). Briefly, cells (1 × 10^5^ per well) were seeded in the upper chambers in serum free RPMI 1640 cell culture medium (in triplicate), and RPMI 1640 medium containing 10% FBS was added to the bottom wells. After 18 hours of incubation, migrated or invaded cells were stained with Diff-Quik Stain Set (Siemens), photographed, and counted manually using Image-pro plus 4.5 (Media Cybernetics, Silver Spring, USA) software.

### Mouse experiment

To examine the *in vivo* effect of LH2 in the growth and metastasis, 344SQ cells expressing scrambled and GATA3 shRNAs were subcutaneously injected (5 × 10^5^ cells per injection) into the flanks of 8 weeks old 129-elite mice (Charles River). Mice were watch three times a week and killed at 4 weeks after injection. No suspicious tumor growth, e.g. tumor regression, was observed during the experiment. At necropsy, both subcutaneous xenograft tumors and the lungs were dissected and fixed in formalin. All mouse experiments and protocols were approved by the Mayo Clinic Institutional Animal Care and Use Committee (IACUC), and the methods were carried out in accordance with the institutional guidelines and regulations.

### RNA extraction and Quantitative RT-PCR

Cells were washed with ice-cold PBS, and subjected to RNA extraction using 700 ul of TRIzol (Invitrogen). mRNA was reverse transcribed using SuperScript IV (Invitrogen). For qPCR reactions, cDNA products were amplified using SYBR Green PCR Master Mix and analyzed using the 7500 fast Real-Time PCR System (both from the Applied Biosystems). mRNA expression levels were normalized to that of the ribosomal protein L32 mRNA by standard curve method.

### Statistics

All statistical comparisons between 2 groups were performed using the 2-tailed Welch’s t test. One-way ANOVA was performed to compare multiple experimental groups. P values that are less than 0.05 are considered statistically significant.

### Data availability

RNA sequencing data and relevant data are available from the authors upon request.

## Results

Our previous studies have shown that knockdown of GATA3 and its co-factor FOG2 suppresses spontaneous metastasis of 344SQ lung adenocarcinoma cells in syngeneic models of metastasis^19,20^. To determine whether FOG2 and GATA3 commonly regulate transcriptome changes to drive metastasis, we performed RNA-sequencing for 344SQ cells that express control scrambled shRNAs or shRNAs against FOG2 or GATA3. Consistent with our previous findings, knockdown of GATA3 significantly reversed EMT-related gene expression changes, as determined by increased expression of epithelial genes and decreased expression of mesenchymal genes (Supplemental Fig. 2A). On the contrary, knockdown of FOG2 had weaker or even opposite effects on their expression (Supplemental Fig. 2A), suggesting that the changes of these EMT-related genes may not be required for metastasis driven by the FOG2/GATA3 complex.

Moreover, our sequencing results revealed that the mRNA expression levels of more than 700 genes were commonly changed upon the knockdown of FOG2 and GATA3 (Supplemental Fig. 2B). Among the common FOG2- and GATA3-regulated genes, LH2 and LH3 were the most attractive for us, because recent results from us and others have shown that LH2 exerts a pro-metastatic function and may have a therapeutic value^7–10^. Sequence analysis showed that both LH2 and LH3 gene promoters contained putative binding sites for GATA3 (Fig. 1A,B), suggesting that they may be direct transcription targets for GATA3. In support of this idea, chromatin immunoprecipitation (ChIP) assays showed that GATA3 bound to multiple candidate GATA3-binding consensus sequences on both LH2 and LH3 promoters (Fig. 1A,B), and knocking down GATA3 inhibited the expression of both LHs (Fig. 1C) and inhibited their promoters’ transcription activities (Fig. 1D). As additional controls, we performed ChIP assays for several other randomly selected GATA3-regulated genes, including SEMA4A, PDE4B, PIK3CD, and E030019813RIK. The results showed that GATA3 also bound to the promoter of SEMA4A (Fig. 1E), but not the other three genes (Fig. 1F), suggesting that GATA3 regulates gene expression changes through both direct and indirect mechanisms. Therefore, it is likely that GATA3 simultaneously regulates multiple transcription targets, which in turn collectively mediate its biological functions.

**Figure 2:**
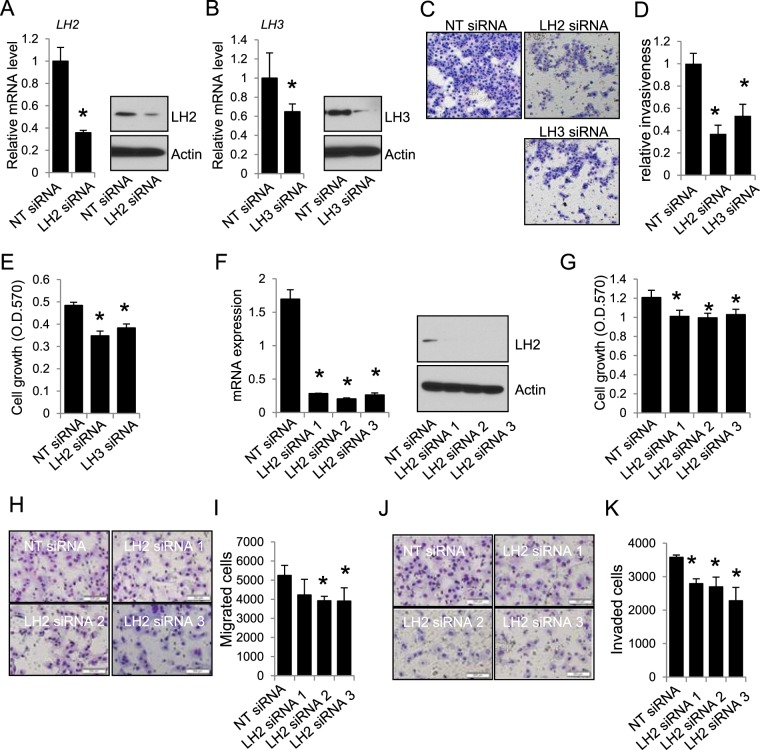
Knockdown of LH2 or LH3 inhibits migration and invasion. (**A**) qPCR and western blotting for 344SQ cells transfected with control non-targeting (NT) or LH2 siRNA. (**B**) qPCR and western blotting for 344SQ cells transfected with control non-targeting (NT) or LH3 siRNA. (**C**) Microscopic images for the 344SQ transfectants that invaded through matrigel-coated transwells. (**D**) The number of invaded cells in (**C**) were counted and normalized to that of NT siRNA transfectants, which was set as 1.0. (**E**) MTT assay for 344SQ transfectants. (**F**) qPCR and western blotting for HCC827 cells transfected with NT or LH2 siRNAs. (**G**) MTT assay for HCC827 transfectants. (**H**) Microscopic images for HCC827 transfectants that migrated through non-coated transwells. (**I**) The number of migrated cells were counted and expressed as mean + stdv. (**J)** Microscopic images for HCC827 transfectants that invaded through matrigel-coated transwells. (**K**) The number of invaded cells were counted and expressed as mean + stdv. Note: in all figures, *indicates t-test p < 0.05.

**Figure 3:**
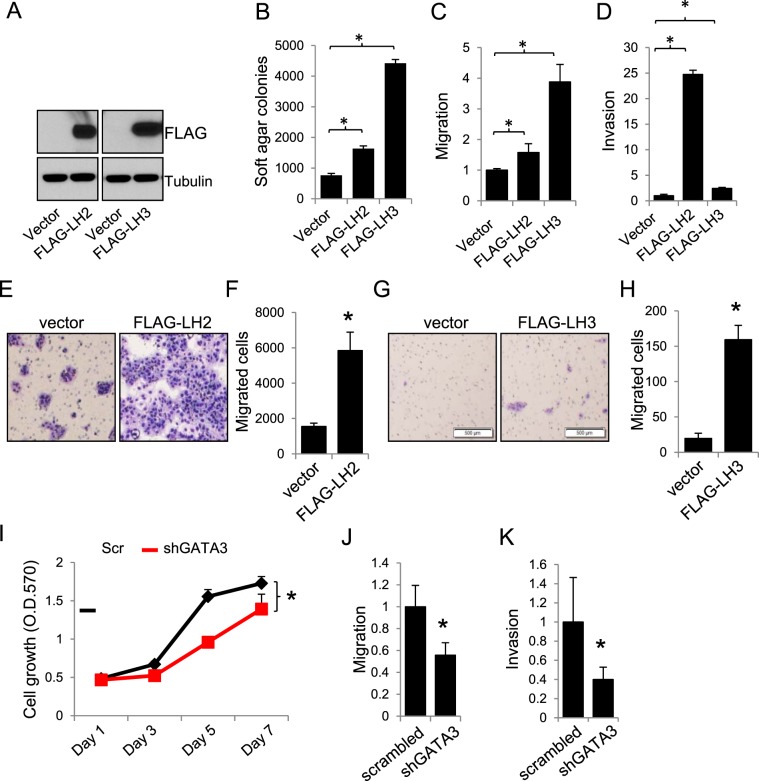
Expression of LH2 or LH3 promotes lung cancer cell growth and migration. (**A**–**D**) Western blotting (**A**), soft agar colony formation, (**B**), migration (**C**), and invasion (**D**) assays for 393 P cells stably expressing FLAG-tagged LH2 and LH3 or empty vectors. (**E**) Microscopic images for the GATA3-depleted 344SQ cells that stably express vector or FLAG-tagged LH2 and migrated through non-coated transwells. (**F**) The number of migrated cells were counted and expressed as mean + stdv. (**G**) Microscopic images for the HCC827 cells that stably express vector or FLAG-tagged LH3 and migrated through non-coated transwells. (**H**) The number of migrated cells were counted and expressed as mean + stdv. (**I**–**K**) MTT (**I**), migration (**J**), and invasion (**K**) assay for 393 P cells transfected with scrambled (scr) or GATA3 shRNAs. Note: in all figures, *indicates t-test p < 0.05.

To determine the role of LH2 and LH3, we knocked down or expressed LH2 and LH3 in multiple mouse and human lung cancer cell lines. We found that knocking down either LH2 or LH3 (Fig. 2A,B) inhibited the invasiveness (Fig. 2C,D) and growth (Fig. 2E) of 344SQ cells. Similarly, knockdown of LH2 in HCC827 cells (Fig. 2F) suppressed their growth (Fig. 2G), migration (Fig. 2H,I), and invasion (Fig. 2J,K). On the contrary, forced expression of either LH2 or LH3 (Fig. 3A) could promote the soft agar colony formation ability (Fig. 3B), migration (Fig. 3C), and invasion of 393 P cells (Fig. 3D). Forced expression of LH2 or LH3 also promoted the migration of other lung tumor cells, including 344SQ (Fig. 3E,F), and HCC827 cells (Fig. 3G,H), precluding any potential specific effects of LH2 or LH3 expression in a single cell line.

**Figure 4:**
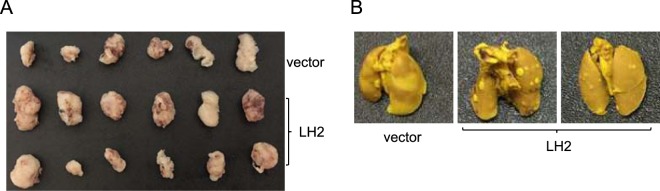
Expression of LH2 promotes xenograft tumor growth and metastasis. (**A**,**B**) The GATA3 shRNA expressing 344SQ lung cancer cells were stably transfected with empty vector or FLAG-tagged LH2. Each transfectant was subcutaneously injected into the flanks of 8 weeks old 129-elite mice, which were killed at 4 weeks after injection. At necropsy, both subcutaneous xenograft tumors (**A**) and the lungs (**B**) were dissected and fixed in formalin.

Consistent with these findings, knocking down GATA3 not only inhibited LH2 and LH3 expression (Fig. 1D), but also suppressed 393 P cell growth (Fig. 3I), migration (Fig. 3J), and invasion (Fig. 3K).These results suggest that LH2 and LH3 may be functionally redundant as the downstream mediators of GATA3, though they may differ in terms of extents in particular assays (Fig. 3B–H). More importantly, stable expression of LH2 alone was sufficient to promote xenograft tumor formation ability and to restore the spontaneous metastatic capacity of GATA3-depleted 344SQ cells (Fig. 4A,B and Table 1), suggesting that the loss of LH2 expression in GATA3-depleted cells (Supplemental Figs 1 and 2) was critical for suppressing tumor growth and metastasis. Furthermore, mining the public Oncomine lung cancer datasets revealed that the expression levels of both LH2 and LH3 were upregulated in lung adenocarcinomas compared to normal lung tissues (Table 2), supporting the notion that these LHs may be useful targets for developing strategies for treating lung cancer.

**Table 1:** Tumorigenicity and metastatic potential of 344SQ_shGATA3 cell ransfectants.

Cell line	Tumorigenicity	Average tumor weight (g)	Lung metastases
Vector	6/6 (100%)	0.59	3/6 (50%)
LH2	12/12 (100%)	0.94	12/12 (100%)

**Table 2:** Lung tumor/normal lung fold changes of LH2 and LH3 mRNAs in the Oncomine datasets.

Genes	Dataset	Garber	Stearman	Selamat	Su	Hou	Talbot
LH2	Fold change	1.996	2.114	2.311	2.335	3.27	3.47
	P value	0.002	3.26e-6	4.27e-13	9.14e-5	4.42e-11	2.14e-11
LH3	Fold change	1.513	1.216	1.28	1.193	1.205	1.508
	P value	0.024	0.005	3.45e-7	0.023	6.89e-4	2.18e-11

## Discussion

There are at least three lysyl hydroxylases (LH1-3) in mammalian cells, and it is known that they have differential enzymatic activities. For instance, although all three LHs catalyze the hydroxylation of lysine residues on collagen, LH2 can act as a specific telopeptide hydroxylase^21^. In addition, LH3 is not only a lysyl hydroxylase but also a glycosyltransferase under certain circumstances^22,23^. Interestingly, despite of these differences, our results show that the manipulation of either LH2 or LH3 has a significant impact on invasion and metastasis (Figs 2 and 3). It is unclear whether LH2 and LH3 exert such redundant functions through differential mechanisms. Therefore, it will be interesting for future studies to determine whether LH2 and LH3 exert such similar functions through their common lysyl hydroxylase activity, or through their distinct enzymatic activities as above-mentioned. Notably, recent studies have shown that both LH2 and LH3 have novel roles as secreted enzymes to remodel extracellular matrix^24,25^, raising another possibility that the two LHs may exert multiple functions associated with their intracellular or extracellular activities. Moreover, it has been shown that different LHs may have distinct substrate specificity and catalyze the hydroxylation of distinct members of collagens^26^, leaving it open to test whether the combined activities of LH2 and LH3 drive metastasis through the modification of multiple collagen isoforms rather than a specific one.

As a reversible cellular process, EMT has been implicated in metastasis in a variety of cancer types, including lung cancer, and many EMT-regulating factors (e.g. transforming growth factor β1, Snail, Slug, Twist, and ZEB factors) are involved in the regulation of metastasis^27–37^. On the other hand, EMT-independent metastasis has also been reported for breast and pancreatic cancers^38,39^. In support of the role of EMT in lung cancer metastasis, our previous studies have shown that GATA3 drives lung adenocarcinoma cell EMT, invasion, and metastasis through the transcriptional repression of miR-200, an epithelial small non-coding RNA that inhibits EMT by targeting ZEB factors^20^. From such an aspect, our findings that FOG2 and GATA3 differentially regulate EMT-related gene expressions suggest that both EMT-dependent and –independent pathways may collectively contribute to lung cancer metastasis, and GATA3 may act as a master regulator of both EMT-dependent and –independent metastasis and serve as a convergence point for the crosstalks between EMT-dependent and –independent pathways.

## Electronic supplementary material

supplementary information
