# Supplementary material for: Lysyl hydroxylases are transcription targets for GATA3 driving lung cancer cell metastasis
Source: Sci Rep. 2018 Aug 9;8:11905. doi: 10.1038/s41598-018-30309-9 (PMC6085355; doi:10.1038/s41598-018-30309-9)
Supplement: Supplementary file 1 — supplementary information [file 41598_2018_30309_MOESM1_ESM.pdf]

Lysyl hydroxylases are transcription targets for GATA3 driving lung cancer cell metastasis

Wei Liu [1, 2, 6], Ting Zhang [1, 6], Lixia Guo [1], Yuanyuan Wang [1, 3], Yanan Yang [1, 4, 5, 7]

[1] Thoracic Disease Research Unit, Division of Pulmonary and Critical Care Medicine, College of Medicine and Science, Mayo Clinic, Rochester, Minnesota, USA

[2] Department of Respiratory, the Second Affiliated Hospital, the Fourth Military Medical University, Xi'an, ShaanXi, China

[3] Department of Respiratory Medicine, East Hospital, Tongji University School of Medicine, Shanghai, China

[4] Department of Biochemistry and Molecular Biology, College of Medicine and Science, Mayo Clinic, Rochester, Minnesota, USA

[5] Developmental Therapeutics and Cell Biology programs, Mayo Clinic Cancer Center, Rochester, Minnesota, USA

[6] These authors contributed equally

[7] Corresponding author. E-mail: yang.yanan@mayo.edu

Fig.1 uncut western blot gels

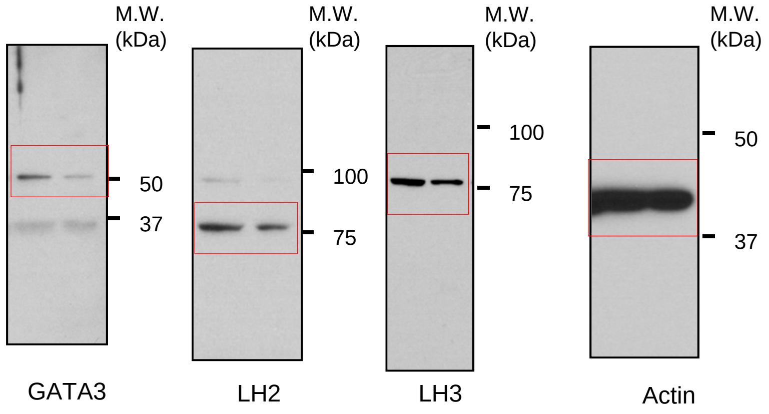

Fig.2 uncut western blot gels

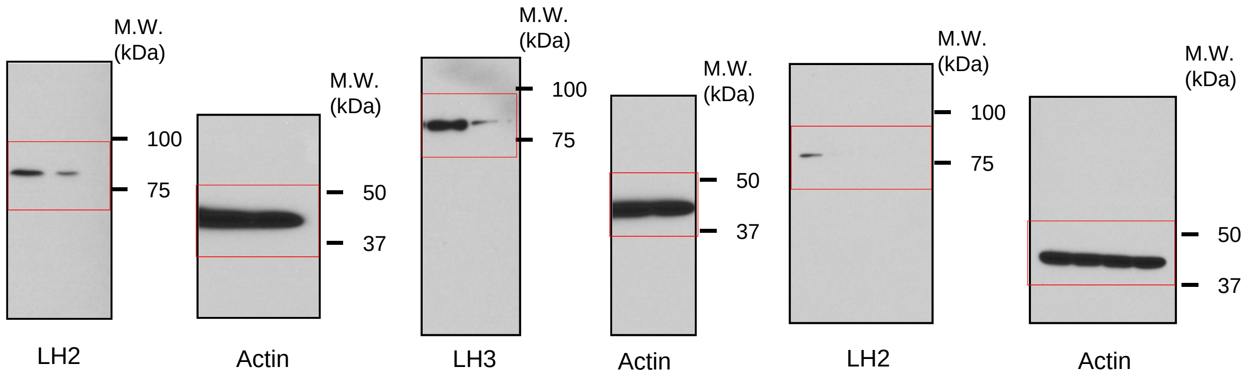

Fig.3 uncut western blot gels

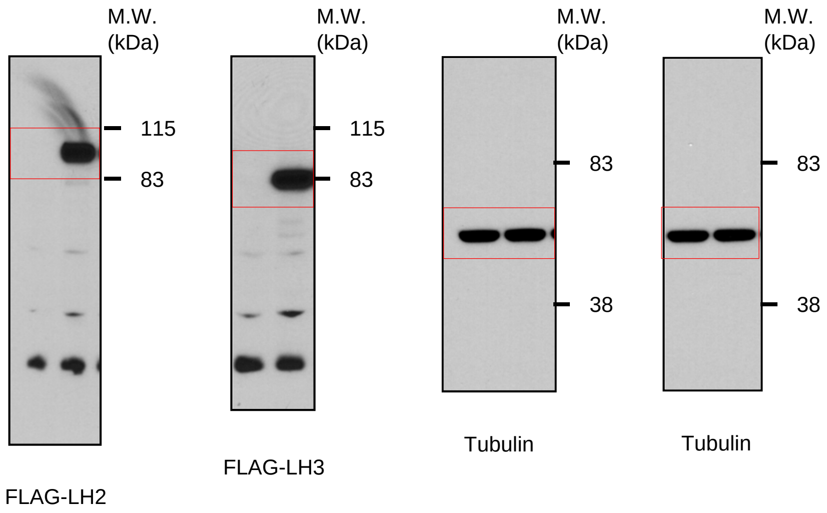

Supplemental Fig.1. Uncut western blot gels.

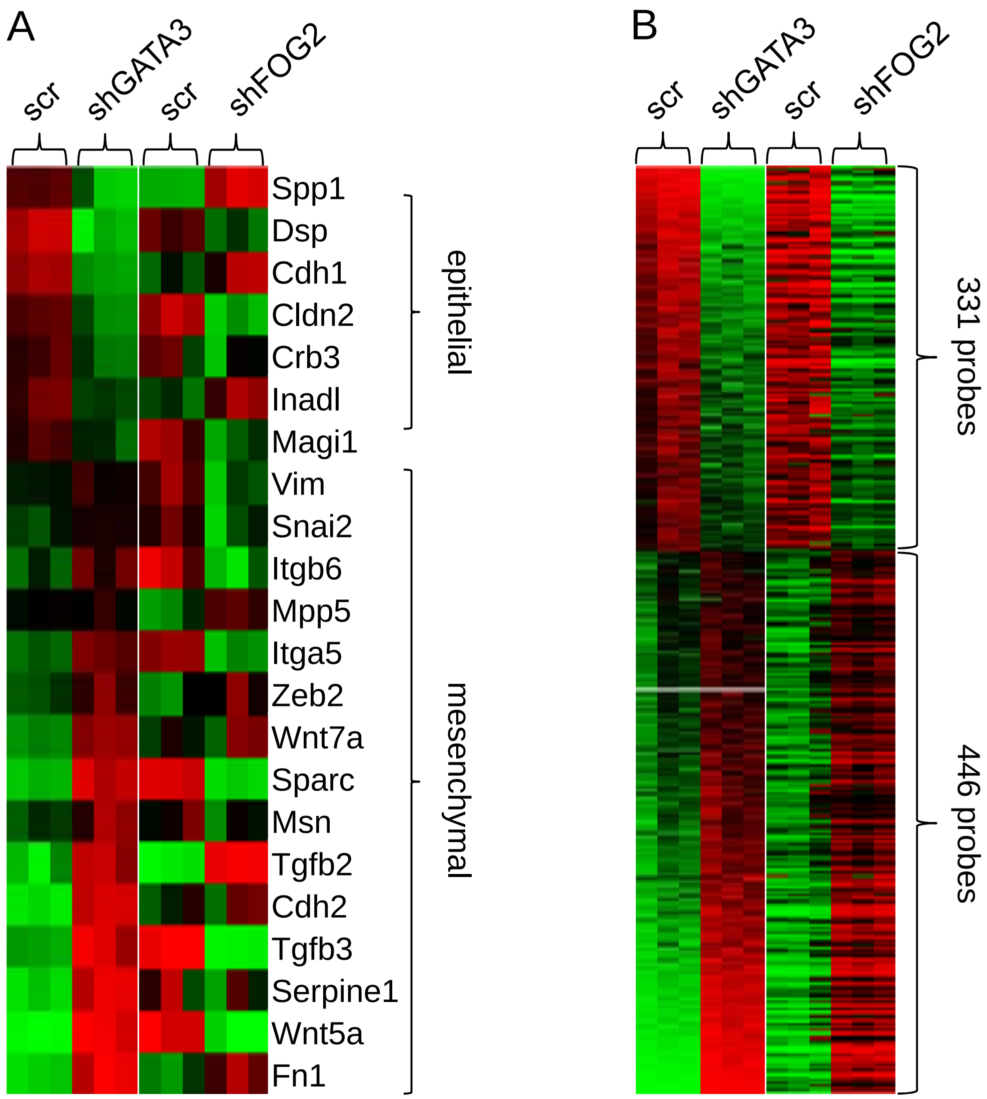

Supplemental Fig.2. GATA3 and FOG2 commonly and differentially regulate gene expression. (A) Heatmap for a list of EMT-related genes that were differentially changed in 344SQ cells expressing FOG2 or GATA3 shRNAs. (B) Heatmap for genes commonly regulated in 344SQ cells expressing FOG2 or GATA3 shRNAs.
